# The interaction between miR165/166 and miR160 regulates *Arabidopsis thaliana* seed size, weight, and number in a ROS-dependent manner

**DOI:** 10.1007/s00425-024-04499-8

**Published:** 2024-08-13

**Authors:** Natalia Pawłasek, Anna Sokołowska, Marek Koter, Krystyna Oracz

**Affiliations:** 1 Department of Plant Physiology, Institute of Biology, Warsaw University of Life Sciences-SGGW, Nowoursynowska Str. 159, 02-776 Warsaw, Poland; 2 Department of Plant Genetics, Breeding and Biotechnology, Institute of Biology, Warsaw University of Life Sciences-SGGW, Nowoursynowska Str. 159, 02-776 Warsaw, Poland

**Keywords:** Hydrogen peroxide, MiRNAs, Reactive oxygen species (ROS), Seed formation, Signaling pathways, Yield

## Abstract

**Main conclusion:**

Our data link the miR165/166- and miR160-mediated regulatory modules to ROS and seed formation. Trade-offs of seed size, weight, and number probably require control of the expression of miR165/166 by miR160, modulation of ROS metabolism by miR165/166, and miR160 abundance by ROS-induced oxidative modifications

**Abstract:**

The cycle of plant life and its yield productivity depends fundamentally on the establishment of the trade-offs of seed size, weight, and number. For annual plants, seed number should simply be a positive function of vegetative biomass and a negative function of seed size and/or weight. However, extensive natural variation within species is observed for these traits, for which an optimal solution is environmentally dependent. Understanding the miRNA-mediated post-transcriptional regulation of gene expression determining seed phenotype and number is crucial from both an evolutionary and applied perspective. Although extensive research has concentrated on the individual roles of miRNAs in plant life, fewer studies have centred on their functional interactions, hence this study aimed to examine whether the module of miR165/miR166 and/or miR160 interactions is involved in forming *Arabidopsis thaliana* seeds, and/or has an impact on their features. Considering that reactive oxygen species (ROS) are among key players in seed-related processes, it was also intriguing to verify if the mechanism of action of these miRNAs is associated with the ROS pathway. The plant material used in this study consisted of flower buds, green siliques, and freshly harvested seeds, of wild type (WT), and STTM165/166 and STTM160 × 165/166 mutants of *A. thaliana* plants which are powerful tools for functional analysis of miRNAs in plants. The novel results obtained during physiological phenotyping together with two-tailed qRT-PCR analysis of mature miR165, miR166, miR160, and spectrofluorimetric measurement of apoplastic hydrogen peroxide (H_2_O_2_) for the first time revealed that interaction between miR165/miR166 and miR160 may regulate seed size, weight and number in ROS-dependent manner.

## Introduction

Plants depend basically on the establishment of seeds. Many signaling pathways and mechanisms regulate seed formation, which ultimately results in a range of variations in seed size, weight, and number. Reactive oxygen species (ROS) and phytohormones are among the major regulators of plant development, including seed-related processes (Oracz and Karpiński 2016). The increased ROS level may lead to oxidative modification of some proteins, mRNA, and microRNAs (miRNAs) (Oracz et al. 2007; El-Maarouf-Bouteau et al. 2013; Huang et al. 2019). In plants, a strong correlation between the redox status of the cell and miRNA production, and/or expression was demonstrated (Ramakrishnan et al. 2022). Many H_2_O_2_-responsive miRNAs were identified in *Oryza sativa*, *Triticum aestivum*, and *Brachypodium*. However, our current understanding of the possible interplay between miRNAs and ROS during seed formation is still very poor.

The short non-coding RNAs (approx. 20–24 nucleotides long), such as miRNAs play a crucial role in the regulation of gene expression at the post-transcriptional level during plant development. In most cases, the mechanism of action of miRNAs requires interaction with 3′UTR, resulting in degradation of target mRNA and/or repression of translation. There are also pieces of evidence that miRNAs may interact with 5′UTR and/or gene promoters. The interaction of miRNAs with target mRNA depends on the abundance of miRNA molecules and target mRNAs, the affinity of miRNA–mRNA interactions, as well as on the location of particular miRNA molecules in cellular compartments (O’Brien et al. 2018). MiRNAs can be secreted into extracellular fluids and transported to target cells via vesicles, such as exosomes, or by binding to proteins, including ARGONAUTES (AGO). A common consequence of cell-to-cell movement of miRNAs is their concentration gradient that directs cell patterning (Zhan and Meyers 2023). Interestingly, the recent results published by Meng et al. (2022) also point to a possible interaction between AGO1-dependent intron splicing and miRNA-mediated target recognition.

Among many miRNAs, the biological functions of miR165/166 and miR160 in some aspects of plant growth and development have already been studied. It has been shown that miR165/166 regulates shoot apical meristem (SAM) maintenance, xylem patterning, embryo formation, and the establishment of leaf polarity (Merelo et al. 2016; Ramachandran et al. 2018). The role of miR165/166 in auxin (IAA) and abscisic acid (ABA) metabolism and/or signalling pathways was also demonstrated. The interplay and regulatory circuit between miR165/166 and miR160, and its effect on leaf polarity and drought tolerance in *A. thaliana* was also investigated. It was demonstrated that miR160-directed regulation of IAA response factors (ARFs) contributes to leaf development via IAA signaling genes, whereas miR165/166-mediated *HD-ZIP III TFs* (*HOMEODOMAIN LEUCINE ZIPPER CLASS III TRANSCRIPTION FACTORs*) regulation confers drought tolerance through ABA signaling (Yang et al. 2019). Interestingly, the functional interaction between these miRNAs and endogenous factors in other stages of plant development has not yet been investigated. Taking into account the importance of the role of ROS in seed biology, IAA and ABA during the first steps of seed development, and that miR165/166 together with miR160 are involved in IAA- and ABA-mediated pathways it was intriguing to verify: (i) if these miRNAs are involved in the regulation of seed formation and (ii) if their mechanism of action is associated with the ROS pathway. In this study, STTM (short tandem target mimic) mutants were used as a plant material. STTM developed from Target Mimicry (TM)8, is an effective approach for knocking down miRNAs. This technology has been successfully employed to down-regulate numerous small RNA families in many plant species including *A. thaliana*, demonstrating that it is a powerful tool for the functional analysis of miRNAs in plants (Tang et al. 2012; Peng et al. 2018; Yang et al. 2019). A two-tailed qRT-PCR (ang. quantitative Real-Time Reverse Transcription PCR) as a precise method for highly accurate miR165, miR166, and miR160 quantification, and a spectrofluorimetric measurement for determination of apoplastic H_2_O_2_ production was applied (Schopfer et al. 2001; Androvic et al. 2017). This study for the first time links the miR165/166- and miR160-mediated regulatory modules to ROS and proposes the existence of possible feedback regulation between miRNAs and ROS during seed formation.

## Materials and methods

### Plant materials and growth conditions

The *A. thaliana* (ecotype Columbia-0) wild type (WT), and STTM mutants STTM165/166 and STTM160 × 165/166, provided by Prof. Zhanhui Zhang from Henan Agricultural University, were used as plant material. The detailed characteristics of the transgenic lines of STTM, mutants and a molecular verification of the functionality of the constructs were already published by Tang et al. (2012) and Yang et al. (2019). Plants were grown in soil mixed with perlite (4:1, v:v), in controlled conditions in the growth chamber, at the long-day photoperiod (16 h, light/8 h, darkness), at 22 °C.

### Characteristics of seed phenotypes

The observations of plant material and measurement of seed size were done using the digital magnifying system TARAGNO FHD TREND.

### RNA extraction, cDNA synthesis, and detection of mature miRNAs using two-tailed qRT-PCR

The total mRNA from seeds was extracted using the CTAB method (Stawska and Oracz 2019), while extraction from buds and siliques was performed using GeneJET Plant RNA Purification Mini Kit (Thermo Scientific) according to the manufacturer protocol. Approx. 100 mg of freshly harvested dry, mature seeds, buds, and siliques were used per sample. The components of qScript Flex cDNA Synthesis Kit (Quantabio) and the manufacturer protocol were used for the miRNA’s related cDNA synthesis, while Revert-Aid H minus First cDNA Synthesis Kit (Thermo Scientific) was used further for *ACT7* analysis. The detection of mature miR160, miR165, and miR166 was analyzed by the two-tailed qRT-PCR method (Androvic et al. 2017). The mRNA samples were diluted 10×, to obtain a final working concentration of 20 µg/ml. The RT (reverse transcription) reaction mixture contained 10 ng of total RNA, 1 × RT buffer, 0.05 µM RT primer, 1 µl GSP enhancer, and 0.5 µl RT enzyme. RT reactions were incubated in PCR tubes placed in thermocycler Nexus (Eppendorf) for 1 h at 42 °C, for 5 min at 85 °C, and then held at 4 °C.

The qRT-PCR was performed in a total reaction volume of 10 µl containing Luminaris SyberGreen Master Mix (Roche), 0.4 µM forward and reverse primers, and 0.4 ng of diluted cDNA. Sequences of the miRNA oligonucleotides were obtained from the miRBase Release 21 (www.mirbase.org). The secondary structure of the two-tailed RT primers was predicted using the UNAfold web server (http://unafold.rna.albany.edu/) and RNAfold WebServer (http://rna.tbi.univie.ac.at/). The qRT-PCR primers were designed using the Primer3 program (https://primer3.ut.ee/). Sequences of two-tailed primes used for cDNA synthesis and qRT-PCR analysis are presented in Table 1.

**Table 1:** Sequences of two-tailed and qRT-PCR primers

Gene	NCBI accession no	Mature miRNA	Sequences (5′→3′) of two-tailed primes	L/R	Sequences (5′→3′) of qRT-PCR primers
*MIR160*	AT2G39175	ath-miR160a-3p	CCTCATACGCACTACTAACGACCAGAGCTAGAGAACCTAGCTCACCCACTCAACCTCTTATGC	L	GCACTACTAACGACCAGAGC
				P	GAGGAGCCATGCATAAGAGG
*MIR165*	AT1G01183	ath-miR165a-3p	CTGGTCCGACGCTCACAGACGTAGAGAACCTACGTCAACAATACACACAAAGGGGGA	L	CGCTCACAGACGTAGAGAAC
				P	TCGGACCAGGCTTCATCC
*MIR166*	AT2G46685	ath-miR166a-3p	CCTGGTCCGATAATTTTAAAACTGCGCGCGCGCGCGCGCGCAAGGGGAA	L	GATAATTTTAAAACTGCGCGCG
				P	CCAGGCTTCATTCCCCTTG
*MIR156*	AT2G25095	ath-miR156a-5p	TCTTCTGTCAACCTAACATGCTCTCCAGGTACAGTTGGTACCTGTCTCCTCATAACCAGTGCTC	L	CATGCTCTCCAGGTACAGTT
				R	GTGAGCACTGGTTATGAGGA
*MIR852*	AT4G14504	ath-miR852a	CGCTTATCTTACTCGAACATCTCTCCAGGTACAGTTGGTACCTGTCTCCTCATAACCACAGAAC	L	ACTCGAACATCTCTCCAGGT
				R	GCGCCTTAGTTCTGTGGTTA

The reactions were performed in triplicates and incubated in LightCycler’96 (Roche), and its steps and conditions used were as follows: pre-incubation: (95 °C/10 min) × 1 cycle, amplification: (95 °C/1 min, 58 °C/30 s, 72 °C/1 min) × 45 cycles, melting: (95 °C/5 s, 70 °C/1 min, 95 °C/1 s) × 1 cycle, cooling: (40 °C/30 s) × 1 cycle. Reaction specificity was assessed by the melting curve. The relative expression level was calculated relative to *ACT7*, miR156, and miR852*.*

### Determination of apoplastic H_2_O_2_ content

The content of apoplastic hydrogen peroxide (H_2_O_2_) was determined using the spectrofluorimetric method in the presence of scopoletin solution (7-hydroxy-6-methoxy-coumarin) previously described by Schopfer et al. (2001) and adapted to analyses in this study. The measurement was performed in four biological replicates. Freshly collected 30–35 mg of plant tissue (buds, siliques, dry seeds), were pre-incubated in 0.5 ml 20 mM K-phosphate buffer (pH 6) for 15 min, at 25 °C, with gentle shaking, in darkness. After that, the buffer was removed, and tissue was incubated for 1 h in the presence of 500 µl of 10 mM scopoletin solution (in 20 mM K-phosphate buffer, pH 6) and 10 µl of peroxidase enzyme (POX 100 U/ml), in the same light and temperature conditions as during pre-incubation. The blank sample was containing MiliQ water instead of the plant tissue. On the 96-hole microplate (Greiner 96 F-Bottom) 250 µl of the reaction mixture from each sample was loaded, and the measurement of fluorescence was performed using plate reader CLARIO Star Plus (BMG Labtech). The H_2_O_2_ content was expressed as µM min^−1^ gFW^−1^.

### Statistical analysis

The obtained results were analyzed using Statistica 13.1 Software (Statsoft, Cracow, Poland). The ANOVA analysis and Duncan’s post hoc test were used to determine homogeneous groups in qRT-PCR analysis of WT dormant *A. thaliana* (Col-0) buds, green siliques, and dry seeds (*P* ≤ 0.05). The mean values were calculated, and statistically significant differences were evaluated using ANOVA analysis followed by Tukey’s HSD post hoc test for H_2_O_2_ determination (*P ≤ 0.05; **P ≤ 0.01). Standard deviation (± SD) was also provided to indicate the variations associated with the particular mean values.

## Results and discussion

### MiR165/miR166 are involved in the regulation of seed size, weight, and number

The features and number of the seeds are regulated through multiple pathways including those induced by phytohormones and involving the miRNA (Zhang et al. 2023). It was previously found that the STTM160 mutant of bread wheat was characterized by significantly smaller grain size, and lower grain weight, while STTM165/166 showed reduced seed numbers (Wang et al. 2018). In the present study is shown that mutant STTM165/166 of *A. thaliana* generated two times fewer seeds in comparison with WT and STTM160 × 165/166 plants. However, the average weight of 1000 seeds of the STTM165/166 was higher at about 11% and length at about 13% in comparison with the WT and others (Table 2). The detailed analysis of seed size indicated also that the seeds in the population of STTM165/166 were not uniform in length as 65% of seeds were of a size similar to WT (approx. 64.5–85 px) while approx. 35% were much larger with the length for about 86–102 px. The width of both groups of seeds was similar (approx. 44.2 px). Interestingly, the size and weight of seeds of STTM160 × 165/166 were similar to WT. These novel data indicate that the miR165/166xmiR160 regulatory module is essential for seed formation (number) and features (size, weight) and that miR160 appears to play an opposing role to miR165 and miR166 in this process.

**Table 2:**
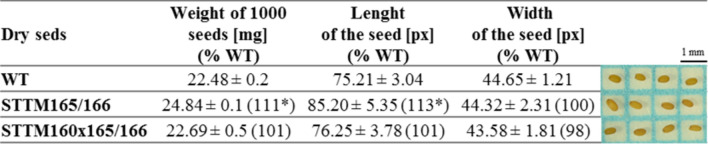
Characteristic of phenotypes of dry (freshly harvested) seeds of WT, and STTM165/166, STTM160 × 165/166 mutants of *A. thaliana*

The results of the measurements of length and width present the mean from 50 randomly chosen seeds of each type ± SD; * indicates the significant differences for *P* ≤ 0.05

### Interplay between miR165/166, miR160, and H_2_O_2_ control seed formation

**Fig. 1:**
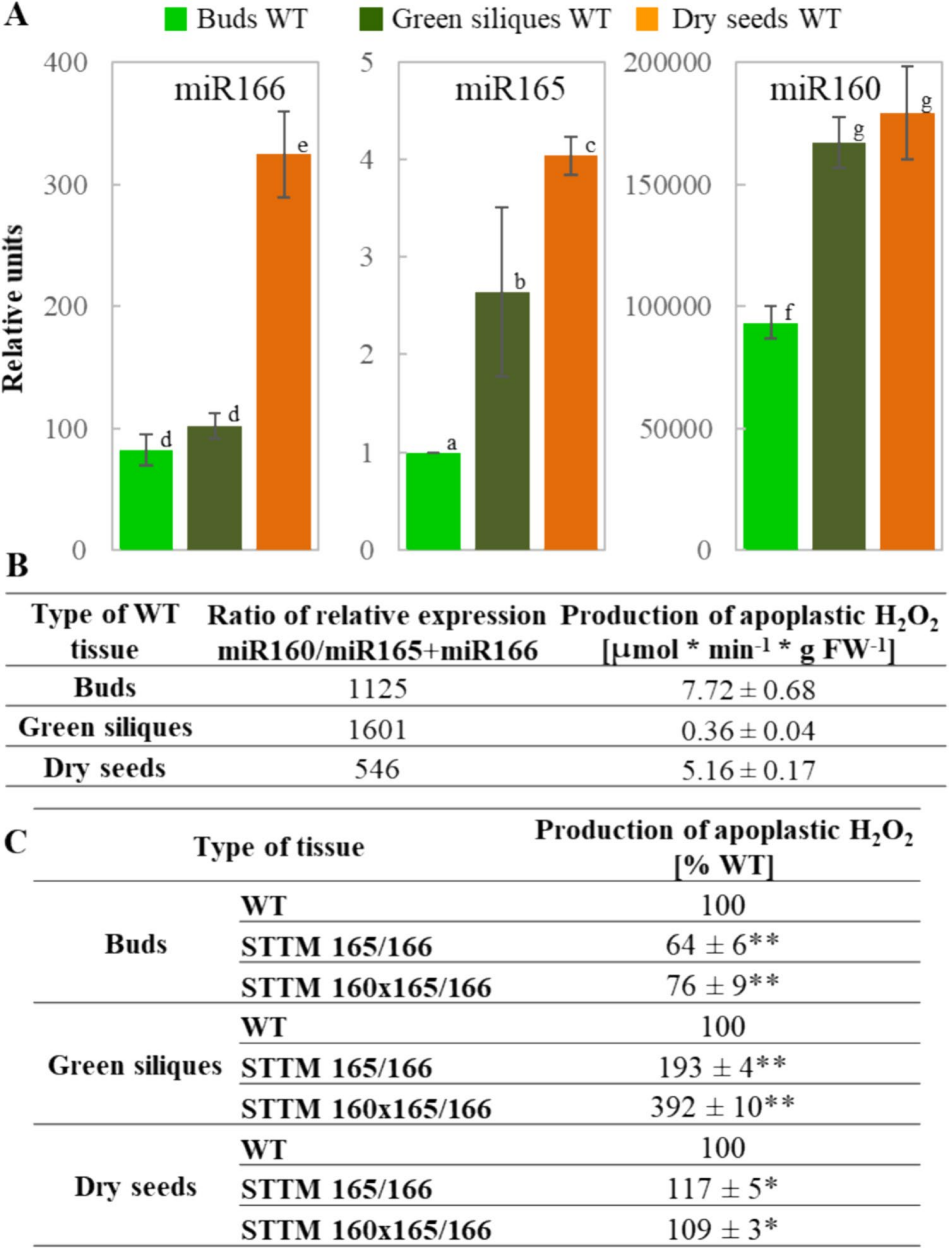
**A** Results of two-tailed qRT-PCR analysis of expression of mature miR166, miR165, miR160. **B** Production of apoplastic H_2_O_2_ in buds, green siliques and dry (freshly harvested) seeds of WT *A. thaliana*. The transcript level was normalized to three reference genes: *ACT7*, miR156, and miR852, and to the internal control, which was the expression of miR165 obtained in buds. The production of apoplastic H_2_O_2_ is expressed in μmol min^−1^ gFW^−1^. Three biological and two technical replicates were performed. The bars show the relative units ± SD, letters a–g indicate homogeneous groups for *P* ≤ 0.05. **C** Comparison of the production of apoplastic H_2_O_2_ in buds, green siliques, and dry (freshly harvested) seeds of WT, STTM165/166 and STTM160 × 165/166 *A. thaliana*. The amount of H_2_O_2_ in a particular type of tissue of WT plants was considered 100%. Three biological and two technical replicates were performed. Results are presented as mean % ± SD; * and ** indicate the significant differences for *P* ≤ 0.05 and *P* ≤ 0.01, respectively

The two-tailed qRT-PCR analysis revealed expression patterns of miR165, miR166, and miR160 in buds, green siliques, and dry (freshly harvested) seeds of WT *A. thaliana* (Fig. 1A). Among the three analyzed miRNAs, miR160 was characterized by the highest relative expression, miR166 by lower, and the miR165 by the lowest in all investigated tissues. These miRNAs expression varied also between the tissues, as the lowest expression was detected in buds, then higher in green siliques, and the highest in dry seeds (Fig. 1A). The expression of miR165 and miR166 was much lower suggesting that miR160 may have a superior role over miR165 and miR166 and may control their abundance. This hypothesis is supported by the results published by Yang et al. (2019) who demonstrated that the expression of miR160 was slightly reduced in STTM165/166 single mutant and that of miR165/166 slightly increased in STTM160 single mutant compared to WT. The authors also showed that, upon comparison of the miRNA abundance with the respective parental lines, the expression level of miR160 was found to be reduced while that of miR165/166 was enhanced in their double mutants.

As the interaction of particular miRNAs with target mRNA depends on the abundance of miRNA molecules and target mRNAs, it is suggested that the ratio of miR160/miR165 + miR166 expression and ROS level may have an impact on the cellular regulation in a particular type of tissue (Fig. 1B). It was found that the ratio of relative expression miR160/miR165 + miR166 at a range from 546 till 1125 was correlated with high production of apoplastic H_2_O_2_ in buds and dry seeds, while the highest range of 1601 with the lowest in green siliques (Fig. 1B). The correlation between production of H_2_O_2_ in STTM165/166 and STTM160 × 165/166 vs WT depended on the type of tissue. In buds there was a decrease, in siliques and dry seeds an increase (Fig. 1C). Interestingly, the changes in H_2_O_2_ level observed in STTM165/166 mutants seems to be partially reversed in STTM160 × 165/166, becoming similar to the level observed in WT. From the literature it is known that miR165 and miR166 are responsible for the down-regulation of expression of genes encoding HD-ZIP III TFs controlling the expression of *HDG11* (*HOMEODOMAIN GLABROUS 11*) gene, which has an impact on the activity of ROS scavenging enzyme such as superoxide dismutase (SOD) (Jia et al. 2015). Therefore on one side, miR165/166 may regulate ROS metabolism (i.e. increase superoxide radical accumulation and decrease H_2_O_2_ by down-regulation of SOD), while on the other hand, the increased ROS level may lead to oxidative modification of some miRNAs. Hence, it may be possible that the expression of miR165/166 is controlled by miR160, while the abundance of miR160 is modulated via oxidative mechanism indicating the existence of possible feedback regulation between miRNAs and ROS during seed formation.

## Data Availability

All data supporting the findings of this study are available within the paper. The gene sequences used in transcriptomic analyses can be accessed at https://www.ncbi.nlm.nih.gov/.

## Abbreviations

IAA: Auxin
ROS: Reactive oxygen species
STTM: Short tandem target mimic, approach for knocking down miRNAs
